# Detachable-dissolvable-microneedle as a potent subunit vaccine delivery device that requires no cold-chain

**DOI:** 10.1016/j.jvacx.2023.100398

**Published:** 2023-10-16

**Authors:** Theerapat Phoka, Naruchit Thanuthanakhun, Peerapat Visitchanakun, Narintorn Dueanphen, Nisha Wanichwecharungruang, Asada Leelahavanichkul, Tanapat Palaga, Kiat Ruxrungtham, Supason Wanichwecharungruang

**Affiliations:** a Center of Excellence in Materials and Bio-Interfaces, Department of Chemistry, Faculty of Science, Chulalongkorn University, Bangkok, Thailand; b Mineed Technology, 928 Block 28, Building D, Chulalongkorn 7 Alley, Bangkok, Thailand; c Department of Microbiology, Faculty of Medicine, Chulalongkorn University, Bangkok, Thailand; d Center of Excellence on Translational Research in Inflammation and Immunology (CETRII), Thailand; e The Petrochemistry and Polymer Science Program, Faculty of Science, Chulalongkorn University, Bangkok, Thailand; f Central Chest Institute of Thailand, Nonthaburi, Thailand; g Department of Microbiology, Faculty of Science, Chulalongkorn University Bangkok, Thailand; h Chula Vaccine Research Center (ChulaVRC) and School of Global Health, Department of Medicine, Faculty of Medicine, Chulalongkorn University, Bangkok 10330, Thailand

**Keywords:** Microneedles, Transdermal vaccination, Subunit vaccine, Vaccine stability

## Abstract

Although vaccine administration by microneedles has been demonstrated, delivery reliability issues have prevented their implementation. Through an *ex vivo* porcine skin experiment, we show visual evidence indicating that detachable dissolvable microneedles (DDMN) can deposit cargo into the dermis with insignificant loss of cargo to the stratum corneum. Using ovalbumin (OVA), a model antigen vaccine, as a cargo, the *ex vivo* experiments yielded a delivery efficiency of 86.08 ± 4.16 %. At room temperature, OVA could be stabilized for up to 35 days in DDMN made from hyaluronic acid and trehalose. The DDMN matrix could improve the denaturation temperature of the OVA from around 70–120 °C to over 150 °C, as demonstrated by differential scanning calorimetric analysis. *In vivo* delivery of OVA antigen into the mice's skin via DDMN elicited 10 times higher specific antibody responses compared to conventional intramuscular injection. We envision DDMN as an effective, precise dosing, intradermal vaccine delivery system that may require no cold-chain, offers a dose-sparing effect, and can be administered easily.

## Highlights

• Drug at tip detachable microneedles (DDMN) are developed for transdermal immunization of subunit vaccine.
• Drug at tip DDMN can deliver drug into dermis with minimal accumulation at stratum corneum.
• DDMN has 86% delivery efficiency.
• Model antigen OVA embedded in DDMN is stable at least 35 days at room temperature.
• At the same dose, DDMN produces 10-fold stronger anti-OVA responses compared to IM vaccination.

## Graphical abstract

**Figure:**
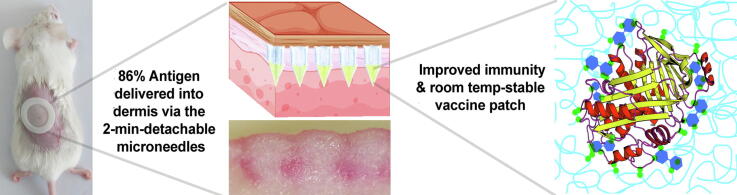

## 1 Introduction

Vaccines serve as one of the most cost-effective strategies for the eradication or reduction of diseases that cause severe illness or death from infection. Vaccines are designed to generate protective immune responses and prevent disease from spreading. To date, the common types of vaccines include live attenuated organisms, killed vaccines, subunit vaccines, viral-like particles, and nucleic acid-base vaccines such as plasmid DNA and messenger RNA vaccines [1], [2]. Subunit vaccines, which consist of purified parts of an organism, are one of the most anticipated new vaccines. This is not only because removing non-essential parts of the organism can reduce the risk of autoimmune or unwanted inflammatory reactions compared to live attenuated or killed vaccines, but also because it lacks genetic material [3], [4]. However, subunit vaccines are inferior to other types of vaccines in terms of immunogenicity and therefore require a formulation with a proper adjuvant [5]. Nevertheless, the use of adjuvants comes with toxicity and adverse side effects [6]. By selecting an appropriate route of administration, it is possible to increase the immunogenicity of the subunit vaccine without incurring additional adverse effects [6], [7]. With abundant antigen-presenting cells, including epidermal Langerhans cells and dermal dendritic cells, at the epidermis-dermis junction, intradermal administration of the vaccine can increase its immunogenicity in comparison to the more commonly administered muscle site [8], [9], [10]. Numerous human studies have proven the validity of this theory [11]. However, not only is it difficult to inject liquid vaccine solution into this thin layer underneath the skin’s surface, but acute pain is unavoidable [10], [12]. Without a skilled professional, it would be easy to miss a site or induce bruising or blistering [13], [14], [15].

Although dissolvable microneedles (DMNs) have long been proposed as a minimally invasive, painless, and easy-to-administer vaccine delivery device with potential benefits in increased immunogenicity and antigen stability to the point where they may not require a cold chain, this device still faces challenges in terms of dose accuracy and delivery reliability [16], [17], [18], [19]. The issues associated with dose accuracy and delivery reliability originate from the prerequisites for two contradictory device characteristics: consistent sharpness of the needles for unfailing skin penetration and rapid dissolution of the needles under a limited amount of water in the skin for complete dose delivery. Needles with a fast dissolution rate in a limited water environment, however, can easily absorb moisture from the air and turn soft, resulting in poor skin penetration. One approach is to use microneedles that can retain the needle’s sharpness but have a slower dissolution rate and are used at a longer administration time. However, this results in other serious issues, such as the unpredictable and difficult-to-notice rebound of the needles from the skin during the extended wearing period and the prolonged opening of the skin at the needle-puncturing points [20], which inhibits the natural rapid resealing process of the skin.

We previously reported on detachable-dissolvable microneedles (DDMN) that enable needles to be detached from the base during administration without relying on the water content of the skin or the rapid dissolution rate of the needle materials [20], [21], [22], [23]. We report here details on 1) the development of the DDMN to have high precision in dosing and reliability in delivering cargoes into the skin; 2) the stabilization of the ovalbumin (OVA) subunit vaccine in the solid DDMN made from biocompatible materials that could alter the denaturation temperature of the protein cargo; and 3) the application of the DDMN to deliver the OVA to the dermis of the mice and the monitoring of their IgG subclass responses. All these three major factors led to our demonstration here of a subunit vaccine in the form of a disposable DDMN patch that not only has a 10-fold higher immunogenicity compared to traditional intramuscular administration but can also be given painlessly in two minutes with accurate dosing and can be kept at room temperature. The paper also includes discussions on the molecular mechanism of improved OVA stability when kept in DDMNs and the biological pathway of increased immunity via DDMN administration.

## 2 Methods

### 2.1 Materials

Sodium hyaluronate (HA; MW 5 kDa and 2000 kDa) was purchased from Baoding Faithful Industry (China). Trehalose, ovalbumin (OVA; from chicken egg white, MW 44.3 kDa), ponceau, and azorubine were purchased from Sigma-Aldrich (St Louis, MO). Anti-OVA (mouse monoclonal antibody clone TOSG1C6) and horseradish peroxidase (HRP)-labeled goat anti-mouse total IgG, IgG1, and IgG2 were purchased from BioLegend (CA, USA). DDMN iron master molds (the circular disk with a diameter of 1.15 cm containing 37 square nail-shaped needles arranged with a tip-to-tip distance of 1150 µm; each needle is nail-shaped with a 250, 250, 270 µm (W, L, H) square column and a 430 µm height of the square pyramid on the top were obtained from Mineed Technology (Thailand). Lint-free polyester sheet assembled with a sticker at the rim was obtained from BOYD Technologies (Thailand) and was subject to gamma sterilization before use. A commercial solution kit for making polydimethyl siloxane silicone rubber molds (food-grade platinum-cured) was purchased from Rungart Resin Company (Thailand).

### 2.1 DDMN fabrication

2.1.1 DDMN mold: A silicone mold was fabricated in-house using the iron master mold following the instructions of the commercial kit for making polydimethyl siloxane silicone rubber mold

2.1.2 OVA-DDMNs*:* The DDMNs were prepared in clean room class A at Mineed Technology Research Laboratory via a mold casting method using a piezo-actuated dispenser to first fill the tip portion of each needle cavity of the DDMN silicone mold with a vaccine-mixed sterile polymer solution (0.2941 μg of OVA in trehalose-HA base for each needle tip). The trehalose-HA base polymer contained HA to trehalose at a 7:3 w/w ratio. The HA used was a mixture of the two MW HA’s, the 5 kDa and the 2000 kDa, at a 6:1 w/w ratio. After drying, each needle was filled with the trehalose-HA mixture with no vaccine. Finally, the lint-free polyester sheet was attached to the base of the needle array. Each DDMN patch contained 10 μg of OVA. DDMN patches with lower doses of OVA (1.0 and 5.0 μg) were also prepared similarly, using appropriate amounts of OVA. All obtained DDMN patches were individually sealed into a plastic blister, put into an aluminum foil pouch, and subjected to OVA quantitation, a delivery efficiency test, and an *in vivo* immunization experiment.

2.1.3 Red dye-DDMNs*:* The red dye-DDMNs were prepared using the same needle structural material (HA and trehalose) and the same preparation procedure as OVA-DDMNs. Each red dye-DDMN patch contains 2.6 μg ponceau and 1.6 μg azorubine at the tip portion of the needles.

2.1.4 OVA-Microneedles with no polyester sheet attached: OVA-microneedles with no polyester sheet attached were prepared using the same process except that the attachment step of the polyester sheet was omitted, and the obtained microneedles were used for the mechanical strength testing, OVA stability experiment, and differential scanning calorimetric (DSC) analysis.

### 2.2 The compressive strength of DDMNs

The mechanical strength testing was performed using the universal testing machine (Shimadzu EZ-S, Japan). The sample was placed on the acrylic plate with the needle facing upward. The plate was then put into the machine containing a compressing probe. The maximum compressive force was set at 200 N. The sample was compressed by a probe during the measurement, and the displaced distance was recorded along with the compressive force.

### 2.3 Stability of OVA in DDMNs

The 1.0 μg OVA-DDMN patches and the 10 μg OVA-DDMN patches were subjected to the stability test by being stored at −20, −4, 25, and 40 °C for 0, 7, 14, 21, 28, and 35 days. OVA was quantified at each time point using indirect ELISA. The test was carried out in comparison to OVA solutions (1.0 and 10 μg OVA in 1 mL of PBS). All experiments were carried out in quintuplicate.

### 2.4 Differential scanning calorimetry

Differential scanning calorimetry (DSC; NETZSCH, STA 409PC) was used to look at how components of the microneedles interacted with each other. The operational conditions were as follows: heating rate of 5 °C/min, from 30 °C to 270 °C, with the flow rate of N_2_ of 50 mL/min. An empty pan was used as a reference.

### 2.5 *Ex vivo* porcine skin delivery efficiency

To examine the skin penetration ability, a red dye-DDMN patch was applied to the *ex vivo* porcine ear skin by manually hand-pressing the patch against the skin for 10 s. The backing of the DDMN was then wet with two drops of water (∼100 μL) using a 1 mL dropper, left for two minutes, then removed. The skin was surgically cut along the line of microneedles that were embedded. The cross-sectioned skin was examined under the stereomicroscope. The dept of skin penetration was then estimated by measuring the distance of red dye from the stratum corneum using stereomicroscopic images.

To quantitatively determine the delivery efficiency, an OVA-DDMN patch was applied to the *ex vivo* porcine ear skin as described above. The removed backing piece was put into 200 mL of PBS buffer to extract the left-over OVA. The surface of the skin after the application was also cleaned with 200 mL of the PBS buffer. The extract was subjected to OVA quantification using an ELISA assay (see below). The delivery efficiency was then determined as follows:

$$\mathrm{Delivery}\;efficiency=\frac{\left(Original\;\;amount\;\;of\;\;OVA\;\;in\;\;the\;\;patch-Amount\;\;of\;\;extracted\;\;OVA\right)}{Original\;\;amount\;\;of\;\;OVA\;\;in\;\;the\;\;patch}\times 100$$

At least three independent replications were conducted for each experiment.

### 2.6 Immunization

The animal experiment was approved by the Institutional Animal Care and Use Committee of the Faculty of Medicine, Chulalongkorn University, Bangkok, Thailand (reference number: 007/2565). Female 6–8-week-old Balb/c mice from Nomura Siam International (Pathumwan, Bangkok, Thailand) were housed at the animal laboratory, Faculty of Medicine, Chulalongkorn University, in an isolated clean room held at 25 ± 2 °C, with a relative humidity of 65–––75 %. The mice were acclimatized for 3 weeks before starting the experiment.

Mice were randomly divided into 6 groups (5 mice/group) as follows: 1.0 μg OVA intramuscular injection (1.0 μg IM), 1.0 μg OVA-DDMN (1.0 μg DDMN), 10 μg IM, 10 μg DDMN, normal saline IM, and blank DDMN. Immunization was done three times for each mouse, on days 0, 14, and 28, according to the previous work [24]. Retro-orbital blood collection was done the same day prior to the immunization and on day 42. Blood was centrifuged at 500 g at 4 °C for 5 min, and serum was harvested and frozen at −80 °C until subsequent analysis. Prior to the DDMN application, the dorsal hair of each mouse was removed using a hair clipper. The DDMN was applied to the shaved skin by 1) thumb pressing the DDMN patch against the skin for 10 s; 2) wetting the backing of the DDMN with two drops of water (∼100 μL total) using a dropper; 3) massaging the wet patch against the skin for two minutes; and 4) removing the backing from the skin. For IM injection, each mouse was injected with 100 μL of OVA prepared in normal saline in the thigh muscles of the hind limb.

### 2.7 Enzyme-linked immunosorbent assay

To quantitate OVA in the microneedles, the 96-well plate (Nunc, MA, USA) was added with 100 ng of OVA samples and allowed to stand at 4 °C overnight. The plate was rinsed three times with washing buffer (PBS plus 0.05 % w/v tween 20, PBST), and then 100 μL of blocking buffer (PBST + 1 % w/v bovine serum albumin) was added and allowed to sit for one hour prior to removal. Then, each well was incubated with a mouse anti-OVA monoclonal antibody (1:2500). The plate was further incubated with goat anti-mouse total IgG-HRP (1:2000) at 37 °C for 1 h.Then the plate was washed three times with PBST. The antigen–antibody reactivity was determined using TMB substrate (BioLegend) according to the manufacturer’s instructions through the measurement of optical density at 450 nm (OD_450_) spectrophotometrically (Varioskan Flash Spectral Scanning Multimode Reader, Thermo Scientific), with the aid of the standard curve generated from a two-fold serial dilution of OVA standard solutions (diluted in PBS from 2000 to 15.6 μg/ml). The OD_450_ of the sample was subtracted with that of the blank polymer.

To assess the anti-OVA IgG responses, the 96-well plate (Nunc, MA, USA) was coated with 100 ng/well OVA in PBS and incubated at 4 °C overnight. The plate was rinsed three times with a washing buffer, and then 100 μL of blocking buffer was added and allowed to incubate for 1 h prior to removal. The diluted serum (serial dilutions of serum were carried out in blocking buffer) was added into the well (100 μL/well) and incubated at 37 °C for 1 h.The plate was then washed three times with a washing buffer. Goat anti-mouse total IgG-HRP, goat anti-mouse IgG1-HRP, and goat anti-mouse IgG2a-HRP were prepared in blocking buffer and used to detect IgG responses. Each antibody was added to each plate at the concentration specified according to the manufacturer’s instructions. The plate was further incubated at 37 °C for 1 h and washed three times. Then the reactivity was detected by the TMB substrate, as described above. The OD_450_ was corrected by subtraction with that of the BSA-coated wells. Antibody titers were calculated as the serum dilution that provided 50 % of maximal binding.

### 2.8 Statistical analysis

Results were expressed as mean ± SD. Two-way AVOVA with Tukey’s post-hoc test was used to determine statistical difference of OVA contents within and between groups. Mann–Whitney *U* test was used to determine the difference of antibody titer between each group. All statistical analyses were performed using GraphPad Prism 8 software (GraphPad Software Inc., San Diego, CA, USA). A p-value < 0.05 was considered statistically significant.

## 3 Results

### 3.1 DDMN preparation

**Fig. 1:**
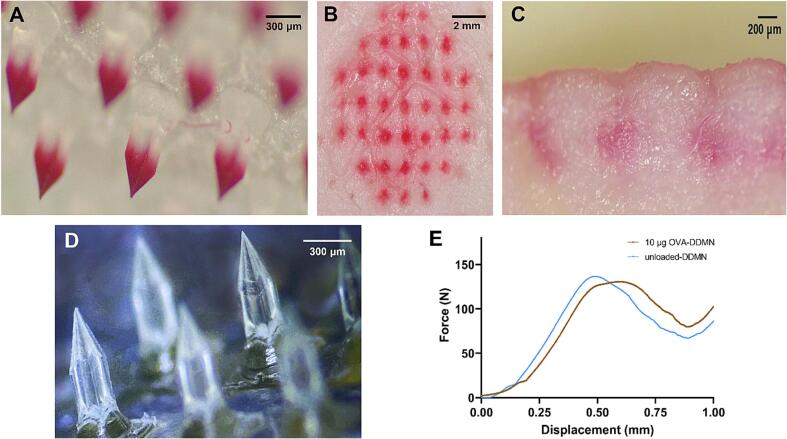
**DDMN morphology and characteristics**. Representative stereomicroscopic images of red dye-DDMNs (**A**); Top view (**B**) and cross-sectioned view (**C**) of porcine skin that was administered with red dye-DDMNs; and OVA-DDMNs (**D**). Compressive strength of the OVA-microneedle (**E**). (For interpretation of the references to color in this figure legend, the reader is referred to the web version of this article.)

The DDMNs could be successfully fabricated using the HA-trehalose blend as the needle material (Fig. 1A). Homogenous monophasic amorphous microneedle was observed. The dimension of the DDMNs was the circular patch with a diameter of 1.15 cm containing 37 needles arranged with a tip-to-tip distance of 1150 µm; each needle is square nail-shaped with a 230, 230, 270 µm (W, L, H) square column and a 420 µm height of the square pyramid on the top. The tip of each needle is sharp. The red dye as a model drug loading was concentrated at the pyramid tip, with very little dye diffusing up to the square column of the needles; there was no dye visible at the base layer (Fig. 1A). These DDMN patches were subjected to *ex vivo* porcine skin penetration test.

After the application, all 37 needles on the patch were able to punch into the porcine ear skin, as observable by the red dots on the skin (Fig. 1B). Cross-sectioned tissue revealed the red color under the skin surface (Fig. 1C). There was no significant red dye accumulated at the stratum corneum, agreeing well with the structure of the needles that red dye was located at the tip portion of the 690 μm long needles. The red spots in the skin tissue spanned from 356 ± 51 to 676 ± 49 μm from the skin surface, implying successful delivery into the dermis.

The OVA-DDMNs could also be successfully fabricated using the same HA-trehalose blend (Fig. 1D). The OVA-DDMNs possessed needles with tips that were obviously less transparent and, to some extent, more yellowish compared to the rest of the needles, implying the deposition of OVA at the tip.

The compressive forces of unloaded DDMN and 10 μg OVA-DDMN were 135 N and 130 N, respectively (Fig. 1E), indicating strong needles that would not break easily.

### 3.2 Degradation of OVA during DDMN fabrication process

**Fig. 2:**
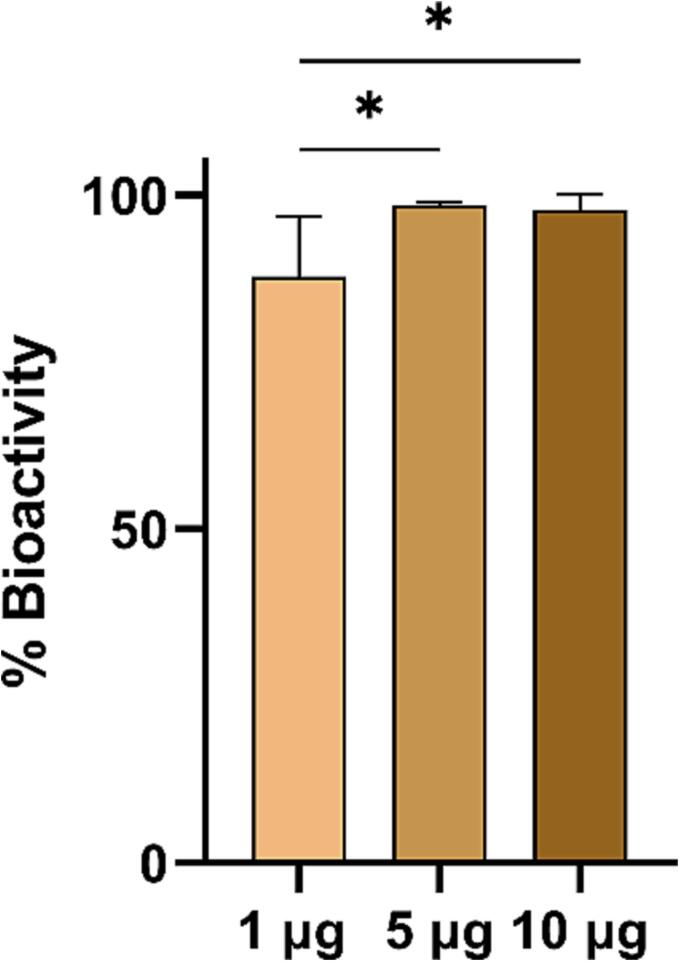
**Relative bioactivity of OVA after DDMN fabrication process.** DDMN patches containing 1, 5, and 10 μg of OVA were prepared and subjected to indirect ELISA analysis to quantify the bioactivity of OVA. The same concentration of fresh OVA solution was used as a reference. A Mann-Whitney *U* test was used to determine the statistical difference (* indicates a significant difference at p < 0.05).

We developed an in-house ELISA to quantify non-degraded OVA (bioactive OVA) in DDMN patches. The prepared DDMN patches loaded with 1, 5, and 10 µg were analyzed (n = 5 each) and compared to the freshly prepared OVA standard solutions. As shown in Fig. 2, the amounts of non-degraded OVA in DDMN patches were 87.82 ± 8.93 %, 98.42 ± 0.479 %, and 98.68 ± 2.41 % for the 1, 5, and 10 µg DDMN patches, respectively. The data suggest that the DDMN fabrication process did not destroy OVA biological activity significantly for the 5 and 10 µg doses. However, some decrease of OVA bioactivity took place during the DDMN fabrication for the 1 µg dose.

### 3.3 Stability of OVA in the DDMNs kept at various temperatures

**Fig. 3:**
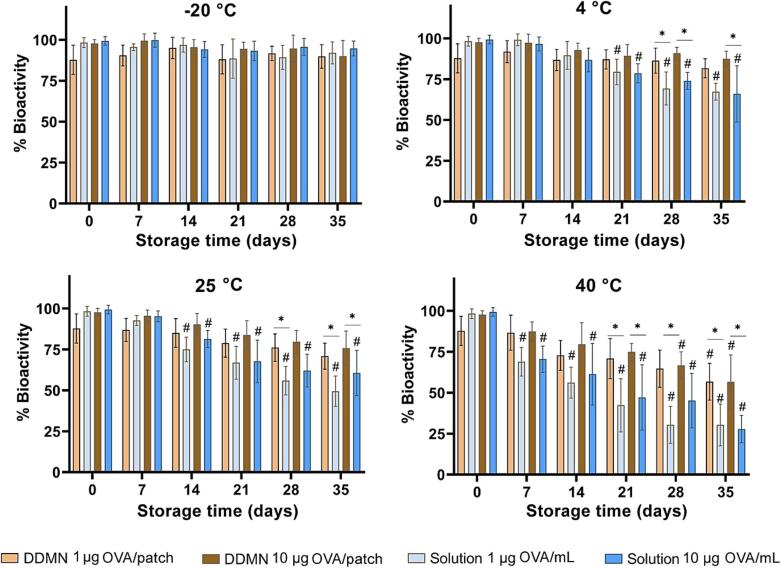
**Stability of OVA in DDMNs**. Bioactivity of OVA in DDMNs and in solutions kept at various temperatures for 35 days, as determined by indirect ELISA. A two-way ANOVA with Tukey’s post-hoc test was used to determine the statistical difference. The ^#^ indicates a significant difference at p < 0.05 between each time point and day 0, and the * indicates a significant difference at p < 0.05 at the same time point.

The biological activity of the OVA in the DDMN patches was compared to that kept in solutions at various temperatures over a 35-day period. As shown in Fig. 3, OVA kept in DDMN showed no significant decrease in biological activity when kept at −20, 4, and 25 °C for the whole 35-day period. However, at 40 °C, the activity started to significantly decrease around the 4th and 5th weeks of storage. In contrast, OVA kept in solution could retain its activity for the whole 35 days only when kept at −20 °C. When kept in solution at 4, 25, and 40 °C, the biological activity started to significantly decrease at the 3rd, 2nd, and 1st weeks, respectively. The result clearly indicates that DDMN helps improve thermal stability of OVA.

### 3.4 Thermal property of pure OVA and OVA mixed with trehalose-HA

To comprehend the enhanced thermal stability of OVA in DDMNs, differential scanning calorimetry (DSC) was used to look into the thermal property of the materials. The technique allows us to visualize the disruption of bonds in the materials through their heat absorption characters, as energy is needed to break the bond. For example, during the melting of trehalose, the breaking of intermolecular bonds that hold the trehalose molecules together in a solid crystalline state should be detectable through the heat absorption at the melting temperature of the material. If the configuration of the OVA changes, the disruption of intermolecular and intramolecular hydrogen bonds and other non-covalent interactions associated with the change should be visualizable through the absorption of heat at the temperatures at which the bond dissociations occur.

**Fig. 4:**
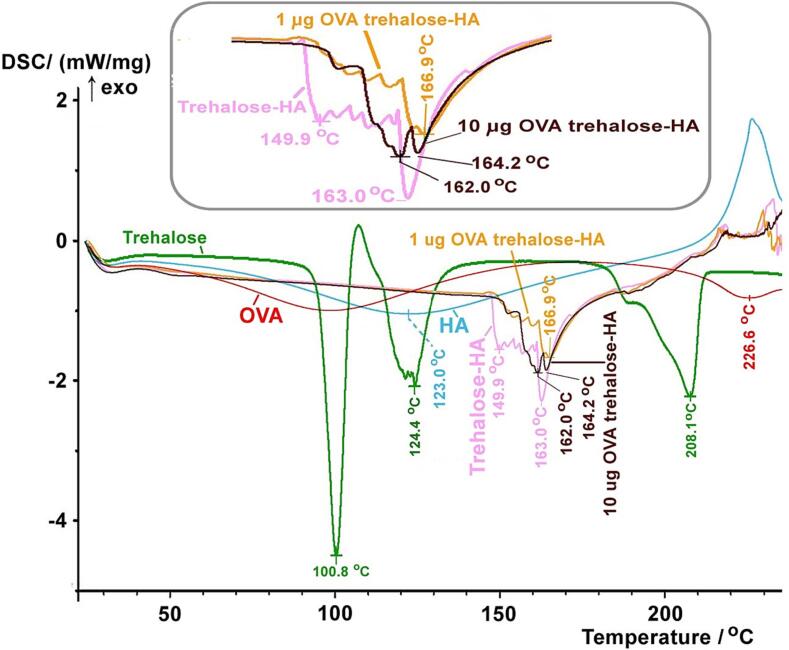
**Thermograms of materials.** DSC thermograms of OVA (red line), trehalose (green line), HA (blue line), trehalose-HA mixture (pink line), 1.0 μg OVA-trehalose-HA mixture (yellow line), and 10 μg OVA-trehalose-HA mixture (dark brown line). The thermograms were obtained at a scan rate of 10 °C/min. The inset shows the expansion at 145–170 °C. (For interpretation of the references to color in this figure legend, the reader is referred to the web version of this article.)

The obtained DSC thermograms of each component (OVA, HA, trehalose), trehalose-HA mixture (represents unloaded needle), 1.0 µg OVA-trehalose-HA mixture (represents DDMN with OVA of 1 µg/patch), and 10 µg OVA-trehalose-HA mixture (represents DDMN with OVA of 10 µg/patch) are shown in Fig. 4.

The three endotherms at ∼ 100, 120–130, and 190–210 °C observed for trehalose (green line Fig. 4), represent the melting of many forms of anhydrous crystalline trehalose, the glass transition of amorphous dihydrate trehalose, and the interchange among various crystalline and amorphous forms of this sugar [25], [26]. The broad heat absorption peak of OVA at 40–170 °C (red line Fig. 4) indicates that this protein starts to change its natural configuration at 40 °C [27], [28]. The very broad heat absorption peaks of HA (blue line Fig. 4), with the maximum at 123 °C, correspond to the glass transition of HA.

The thermogram of the trehalose-HA mixture (pink line Fig. 4) reveals the absence of the previously described characteristic heat absorption peaks of trehalose and HA; instead, the mixture exhibits new heat absorption peaks at higher temperatures (150–170 °C). This suggests that the trehalose-trehalose and HA-HA molecular interactions in the pure compounds have been replaced by more favorable and more stable trehalose-HA interactions, as the new heat absorption peaks of the mixture are at a higher temperature.

The thermograms of the two OVA-trehalose-HA mixtures (the 1.0 and the 10 g OVA, orange, and dark brown lines Fig. 4, respectively) lack the aforementioned characteristic peaks of the three constituents, OVA, trehalose, and HA. The mixtures exhibit new heat absorption peaks at 155–165 °C (inset Fig. 4). This heat absorption at higher temperature indicates that the molecules in this three-component mixture are interacting strongly; a high temperature is needed to break their interactions. The information implies better thermal stability for OVA in the mixture compared to pure OVA.

In short, DSC results indicate strong molecular interactions among OVA and other components in the microneedles, and these interactions are likely responsible for the enhanced thermal stability of OVA in the DDMN.

### 3.5 Delivery efficiency

As described above, we have successfully fabricated OVA-DDMN, in which OVA was concentrated at the microneedle tips. We applied these OVA-DDMNs to *ex vivo* porcine skin and determined the amount of OVA remaining in the removed DDMN backing and at the skin surface. There was no OVA detected on the backing sheet. Approximately 13.92 ± 4.16 % OVA could be reconstituted from the skin surface. Therefore, percentage of OVA administered to the tissue underneath the skin surface was 86.08 ± 4.16 %.

It should be noted here that attempts were made to quantify OVA in the skin tissue. Nevertheless, we could not achieve reliable OVA extraction and detection either for the control (hypodermic injection of a precise dose of OVA) or the OVA-DDMN administered skin.

### 3.6 IgG responses against OVA delivered via DDMNs

**Fig. 5:**
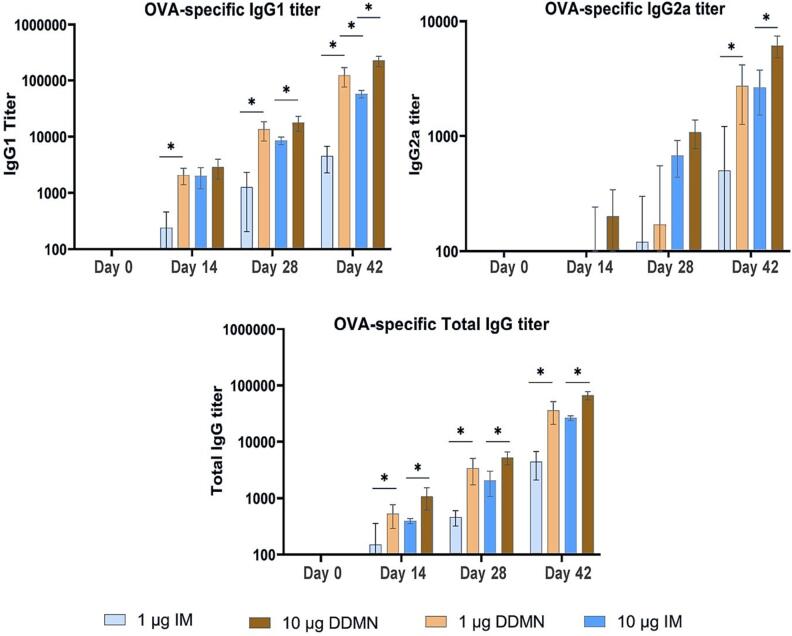
**Anti-OVA IgG responses**. Anti-OVA IgG1, IgG2a, and total IgG were measured on days 0, 14, 28, and 42 after the first immunization. Mice were immunized on days 0, 14, and 21. Titers were determined by ELISA. Results were expressed as mean ± SD. There was no anti-OVA IgG detected in the serum of the control groups, which include blank DDMN and normal saline IM (not shown in the graphs). A Mann–Whitney *U* test was used to determine the statistical difference (* indicates a significant difference at p < 0.05).

The adjuvant-free OVA doses of 1.0 and 10 µg were separately administered to Balb/c mice on days 0, 14, and 28. As shown in Fig. 5, total OVA-specific IgG levels were significantly higher in mice immunized via DDMN than in mice immunized via intramuscular injection (IM). The higher IgG responses of DDMN over IM were observed for both the 1.0 and 10 µg OVA doses, but the discrepancy was more pronounced for the low dose. In addition, the higher levels of both IgG1 and IgG2a subtypes were observed for the DDMN groups. The data suggest that the OVA vaccination via DDMN induced about 10-fold higher IgG responses as compared to via IM. In addition, a higher total IgG titer in the DDMN groups, comparing to the IM groups, at day 14 implies a more rapid induction of immune response via the DDMNs. Lastly, our delivery efficiency experiments revealed that 86.08 ± 4.16 % of OVA in the DDMNs could be delivered into the skin; therefore, the immune response levels observed here were due to the 0.86 and 8.6 µg of OVA delivered into the skin by the 1.0 and 10 µg OVA-DDMN patches, respectively. These dosages are compared to the IM injection doses of 1.0 and 10.0 µg OVA.

## 4 Discussion

Due to its large population of immune cells, which enables an effective immune response with a small amount of antigen, the skin is regarded as a promising target for vaccination [10], [11], [29], [30]. Microneedles are a highly anticipated technology for vaccination via skin; however, delivery reliability has been an issue for quite some time. Here, we demonstrate the use of DDMNs, or detachable microneedles, to resolve this issue so that vaccination through the skin may be performed more reliably and easily.

In this instance, DDMNs with OVA located at the tip could be successfully fabricated using trehalose and HA as needle structural material (Fig. 1D) and they could withstand up to 130 N of compressive force (Fig. 1E), more than enough for normal finger-pressing of microneedle patches against the skin [31], [32], [33], [34], [35]. We also confirmed that the OVA-DDMN fabrication process did not drastically alter the OVA's biological activity (Fig. 2). We did so through the use of an anti-OVA monoclonal antibody that binds to the OVA's three-dimensional epitope. Since binding corresponds directly to the conformation of OVA, the response from the ELISA test developed for this study should indicate the amount of bioactive OVA or the configurationally preserved OVA. In this regard, the DDMN fabrication process decreased OVA biological activity by less than 2 % for the 5 µg and 10 µg OVA-DDMNs and around 10 % for the 1 µg OVA-DDMNs. This is explainable by the previous studies that reported more intense denaturation of OVA in dilute solutions than in more concentrated solutions [36].

The obtained OVA-DDMNs could attain a delivery efficiency of 86.08 ± 4.16 %. This robust efficiency was likely due to the effective and reliable detachment of needles during application and the localization of OVA at the tip of the DDMNs (Fig. 1D), as demonstrated by the absence of OVA in the backing sheet after application. However, 13.92 ± 4.16 % of the applied OVA were detected in the wash of the stratum corneum of the administered skin area. We explain this as follows: although OVA could be localized at the tip, the pressing of the tips against the skin surface during application may have facilitated the transfer of some OVA to the stratum corneum of the skin. It should be noted that this delivery efficiency study is very important because a high and dependable delivery efficiency is essential for the potential use of microneedles in vaccination. The lack of this feature has prevented the utilization of dissolving microneedles in the pharmaceutical industry for quite some time.

Thermal stability experiments revealed an increase in thermal stability for OVA stored in a trehalose-HA DDMN matrix (Fig. 3). The finding is not surprising because increased stability of vaccines stored in dissolving microneedles has been previously reported; for instance, dissolving microneedles fabricated from arginine, calcium heptaglucosate, and sodium carboxymethyl cellulose could maintain the bioactivity and immunogenicity of the influenza vaccine for one year at 25 °C [37], [38]. The nature of the antigen and the composition of the microneedle are crucial variables. Here, the two structural components of the DDMNs, trehalose and HA, likely play a significant role in the stabilization. It has been reported that trehalose is able to protect protein and membrane structures from denaturation [25], [39]. Many organisms biosynthesize trehalose in response to their exposure to heat, dehydration, or freezing [40], [41]. In addition, HA, a natural extracellular glycosaminoglycan of human skin, has also been reported as a material that can help preserve biological structures and cells [22], [42], [43]. The combination of trehalose and HA has been demonstrated to stabilize protein structure during freeze-drying [44].

The speculation that trehalose and HA stabilize OVA through intermolecular interactions is verified with the DSC experimental results (Fig. 4). In brief, the weaker intermolecular interactions observed for each of the three constituents, OVA, trehalose, and HA (their heat absorption peaks are at lower temperatures) disappeared when the three materials were mixed, and stronger interactions emerged (new heat absorption peaks of the mixture are at higher temperatures). Therefore, it is very likely that trehalose assists in stabilizing OVA by forming strong intermolecular bonds that aid in maintaining the protein's configuration via its rigid calm-shell structure. It is possible that binding of trehalose to OVA replace the hydration water at critical amino acid residues of the OVA. This mechanism of water replacement for protein stabilization by trehalose has previously been proposed [45], [46]. In addition, the solid polymeric network of HA that strongly interacts with OVA likely assists in locking the protein's structure, thereby preventing its unfolding. The locking of biomolecular movement in solid HA matrix has previously been proposed [22].

It should be mentioned here that the base line at 40–150 °C of the thermograms of the OVA–trehalose–HA mixtures is not totally flat; a very small slope can be observed (Fig. 4), implying slight heat absorption by the material in this temperature range. We speculate that such a small heat absorption is associated with the small movement of HA chains. It will be interesting to see if HA could be replaced with some other materials to make a totally flat baseline, which should result in a more thermostable OVA in the DDMN.

Although our results demonstrated the thermal stability of the OVA-DDMN patches, the OVA-DDMNs used to immunize mice were produced, stored at 4 °C, and used within two weeks. After gaining an understanding of the duration and potency of immune induction by OVA-DDMNs in this work, the next study on OVA-DDMNs stored at different times and temperatures can be planned and conducted. It should be noted here that in this study we immunized three times, on days 0, 14, and 28, and we determined anti-OVA IgG responses on days 0, 14, 28, and 42. This experimental design was based on the low immunogenicity of the subunit vaccine and the absence of an adjuvant in the study. Previous studies have shown that even three repeats of immunization of non-adjuvanted OVA via microneedle generated a low total IgG titer [24], [47]. Therefore, to make sure that the experiment, which included the control of the IM route, would give a high enough response, a three-immunization scheme was selected.

As described in the result section, the transdermal immunization of mice with the DDMN patch induced faster and more potent immune responses for both IgG1 and IgG2a subtypes comparing to IM injection of the same OVA doses (Fig. 5). IgG1 and IgG2a can serve as surrogate markers for T helper cell types 2 and 1, respectively. As previous studies in mice showed, IgG1 production was induced by Th2-secreted IL4, whereas IgG2a production was induced by Th1-secreted IFN gamma [48], [49], [50]. Since the goal of the vaccine is to induce both routes of immune responses, the higher levels of the two antibody subtypes observed for DDMN delivery as compared to IM delivery may indicate an improvement in the vaccine's overall efficacy by DDMN delivery [51].

DDMNs gave approximately 10 folds higher total immune responses than intramuscular injection at the same doses (Fig. 5). This result agrees well with previous reports, which indicated that transdermal administration offers a superior dose-sparing effect [8], [9], [10], [11]. Several studies have reported improved immunogenicity for the use of microneedles on the OVA model vaccine antigen [52], [53]. DeSimone and colleagues reported that when OVA was administered with CpG adjuvant via 3D-printed microneedles, OVA-specific IgG levels were 50 times greater than with intradermal and subcutaneous injections [54]. Another study found that total IgG reached a plateau after six vaccinations when OVA was administered without adjuvant through sodium hyaluronate-dextran-povidone dissolving microneedles, and the titer was not different compared to subcutaneous injection [24].

The 10-fold dose sparring observed in this study may be a result of not only the intradermal route but also the improved dermal localization of antigen in the dermis. Since the release of antigen encapsulated in the DDMN matrix relies on the dissolution of HA, the antigen will remain at the embedded site for a longer period than when the antigen is injected as a solution. It has been reported that HA can prolong antigen localization at the administration site for up to 74 h [53]. The extended localization of antigen in the dermis leads to a prolonged period of antigen presentation to antigen-presenting cells, hence enhancing the humoral immune response [55]. Additionally, the dissolved HA attracts more water to the area, which facilitates the tissue hydration and better infiltration of immune cells [56]. HA fragments resulted from the HA digestion by the naturally available hyaluronidases in the skin [57] can also bind to the receptors on the toll-like receptors (TLR) 2 and 4 of dendritic cells [58]. All these roles of HA likely enhance the antigen-presenting cell recruitment and thus produce a more robust immune response for the DDMN groups.

Some limitations in this study should be pointed out here. First, the comparison is between IM injection of OVA solution and intradermal embedment of OVA-loaded microneedles. It will be interesting to compare the intradermal embedment of OVA-loaded microneedles with the intradermal injection of OVA solution. Second, it should be noted here that the DDMN used in this study is designed for mouse skin. Therefore, it might not be directly applicable to humans since mice's skin is looser and thinner in the stratum corneum, epidermis, and dermis in comparison to humans [59]. Since humans have much thicker dermis, it might be a good consideration to explore the longer needles.

## 5 Conclusion

In summary, here we demonstrate the use of detachable dissolvable microneedles (DDMN) for intradermal vaccine delivery. First, we demonstrated visually that DDMN can deposit cargo into the dermis with negligible loss to the stratum corneum by using red dye as the cargo. Then, we loaded ovalbumin (OVA), a model antigen vaccine, to the tip of DDMN and examined its delivery efficiency in *ex vivo* porcine skin. The outcome indicated a delivery efficiency of 86.08 ± 4.16 %. These two experiments suggested that the detachable feature of the DDMN, which permits needle detachment from the base during skin administration so that needles can be imbedded in the skin within two minutes of administration, could solve the delivery reliability problem that microneedles have had for a long time. In addition, the use of trehalose and HA as needle structure materials improved the thermal stability of the OVA contained within the needles; for instance, the OVA-trehalose-HA DDMN could be stored at 25 °C for 35 days without losing its biological activity. Through DSC analysis, we also demonstrated that when OVA was complexed with trehalose and HA, the starting denaturation temperature of OVA increased from 40 °C to over 150 °C. Finally, we compared the immunogenicity of OVA-DDMN to that of a conventional intramuscular (IM) injection of OVA solution. We found that the DDMN was able to elicit 10 times more specific antibody responses than the IM.

**Funding**

This work was funded by the National Research Council of Thailand, and the Government Pharmaceutical Organization, Thailand. T.P. was supported by the Second Century Fund (C2F) from Chulalongkorn University.

## Declaration of Competing Interest

Mineed Technology Company owns the detachable dissolvable microneedle patent.

## Data Availability

Data will be made available on request.
